# Adaptive sampling in the Philippine Sea using autonomous profiling floats and FloatCast

**DOI:** 10.3389/frobt.2026.1813102

**Published:** 2026-07-31

**Authors:** Joseph R. Tolone, Trevor W. Harrison, Zoltan B. Szuts, Derek A. Paley

**Affiliations:** 1 Department of Aerospace Engineering and Institute for Systems Research, University of Maryland, College Park, MD, United States; 2 Applied Physics Laboratory, Ocean Physics Department, University of Washington, Seattle, WA, United States

**Keywords:** adaptive ocean sampling, echo state network, Lagrangian particle model, mapping error, profiling floats, real-time control

## Abstract

This work details a method for manipulating the horizontal position of free-drifting ocean-profiling floats using vertical depth control. Profiling floats are often used to study oceanographic processes of varying spatial and temporal scales. Since deploying floats is logistically demanding and expensive, active floats must collect measurements most relevant to the sampling objectives. To that end, we present the FloatCast framework for managing drift in a fleet of floats via vertical depth control. The FloatCast software selects float dive commands that are anticipated to align best with sampling objectives by combining tools from machine learning, optimization, and feedback control. First, an echo state network is used to generate forecasts of ocean surface flow. These forecasts are used to compute float trajectory predictions for each set of candidate dive commands. The performance of each set of dive commands is then ranked using a mapping error scoring metric, and the highest-ranking command set is provided to the floats for their next dive. Reported here are simulation and real-time experimental results from FloatCast control in the Philippine Sea. The simulation results provide insight into the broader FloatCast methodology; experimental results show that this control framework has promise, though it offers no performance guarantees. Although these floats are inherently underactuated, implementing FloatCast can increase the sampling effectiveness of a float array and, in turn, improve scientific data products.

## 1 Introduction

Understanding oceanographic processes and circulation features is vital for informing environmental policies because of the interconnection between Earth’s oceans, atmosphere, and continents. The Argo Program is a key technology for describing the ocean–climate relationship (Lidström and Wickberg, 2025). This program uses thousands of free-drifting profiling floats distributed globally to periodically measure ocean characteristics (Wong et al., 2020). Similar profiling floats are used to collect regional measurements that compliment typical Argo data and target specific ocean processes. Here, we endeavor to improve the quality of regional measurements by directing floats to dynamically sample areas of greatest interest. In contrast to the standard practice of uniform profiling behavior with passive horizontal drift, we seek to manipulate the horizontal position of floats by using buoyancy control to move them to depths with favorable flow. An adaptive sampling formulation is used so that decisions on vertical depth control are affected by sampling history.

Profiling float data can improve our understanding of environmental processes that are important for climate change and sustainability research. For example, Kubin et al. (2023) used Argo data to study regional heat increase in the Mediterranean Sea. On a global scale, Johnson and Purkey (2024) used Deep Argo data to considerably reduce the uncertainty associated with heating trends in the deep ocean. Marine heatwaves (Holbrook et al., 2020) and the movement of marine plastic debris (Van Sebille et al., 2020) can also be studied using float measurement and trajectory data. In addition, profiling floats may be used to monitor ocean acoustics (Ma et al., 2023). Our research objective is to enhance the efficiency of these regional ocean sampling missions by leveraging local ocean currents to improve the horizontal sample distribution of float arrays.

**FIGURE 1:**
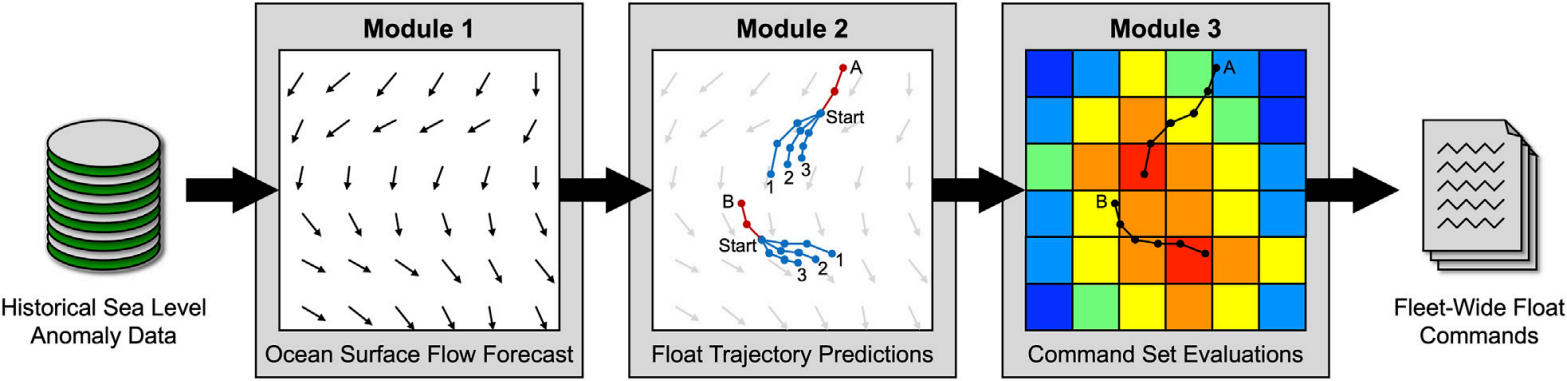
FloatCast consists of three main modules: (1) the ocean surface flow forecaster; (2) the float trajectory predictor; and (3) the float dive command selector. An echo state network is used to produce surface flow forecasts. These are given to the float trajectory integrator, which predicts float trajectories based on a set of candidate dive commands. The performance of predicted float trajectories is evaluated using a mapping error scoring metric. The commands generating the best set of trajectories are sent to the active floats. Float location data is fed back into the system for the next planning cycle.

For a given set of sampling constraints (e.g., mission duration, available floats, environmental factors), the goal is to obtain samples most relevant to research interests. To that end, we built a custom modular software tool, FloatCast, for the intelligent control of a small fleet of floats. This tool maneuvers profiling floats via buoyancy control to obtain measurements that best resolve regional circulation or other processes that occur over days or weeks. The technical approach combines machine-learning techniques for ocean forecasting with a dynamic model of float motion to optimize dive commands given to floats in real time. For our purposes, a dive command consists of a parking depth and duration. This control process relies on the natural movement of ocean currents for horizontal float actuation. A schematic of the FloatCast software is shown in Figure 1.

FloatCast employs an Echo State Network (ESN) to forecast ocean surface flows, which are used to predict float trajectories. ESNs are a data-driven prediction method and have a variety of uses (Sun et al., 2024). Hassanibesheli et al. (2022) used an ESN to forecast sea-surface temperature to predict El Niño Southern Oscillation behavior. Cui et al. (2025) trained a deep neural network to predict sea surface height along with temperature, salinity, and horizontal currents in the upper layers of the ocean. Here, we use an ESN to forecast ocean surface flow and infer float trajectories from these predictions.

As noted above, profiling floats are used extensively in the Argo project (Wong et al., 2020). Argo floats follow fixed dive commands to obtain statistically meaningful observations of ocean changes over decades. Recent studies by Wang et al. (2024), Liu et al. (2025), and Chamberlain et al. (2023a) investigated the best deployment locations for Argo floats to maintain spatial coverage. Wang et al. (2024) and Liu et al. (2025) both use Lagrangian particle models to predict expected float trajectories. Chamberlain et al. (2023b) used a transition matrix prediction method similar to the Perron–Frobenius operator^1^ to predict the global distribution of Argo floats. Grossi et al. (2025) applied machine-learning techniques to predict surface drifter trajectories based on historical data. FloatCast uses a Lagrangian particle model for trajectory predictions because it is computationally lightweight.

Controlling underactuated ocean vehicles is a challenging problem. Recently, Wiggert et al. (2022) used dynamically updated ocean forecasts to plan movements of an underactuated holonomic vehicle. In our work, we consider platforms that have no direct lateral control. Smith and Huynh (2014), Troesch et al. (2018), and Szuts et al. (2023) have addressed the problem of directing the lateral movement of profiling floats using vertical depth control. Smith and Huynh (2014) utilized a path-planning approach in which control decisions were made based on the probability of success. Troesch et al. (2018) used float depth control to make virtual moorings in an effort to keep floats close to a given location. We use depth control and float modeling techniques similar to those of Troesch et al. (2018), but we tailor our methodology to spatial and temporal scales of much larger magnitude—specifically through our techniques for flow forecasting, float trajectory prediction, and command selection. Szuts et al. (2023) constructed the FlowPilot software to use multiple prediction models to compute optimal dive commands and send them to active floats. The FlowPilot software sent FloatCast commands to active floats for the work presented here.

To assess the quality of predicted float trajectories for sets of dive commands, we use mapping error as the scoring metric. This metric is based on prior work by Leonard et al. (2007) and Paley (2007), who used a similar formulation for an ocean sampling problem for underwater gliders. The specific technique comes from the optimal interpolation method, which assimilates real-world samples to infer likely measurement values at predetermined locations (Bretherton et al., 1976; Leonard et al., 2007). The mapping error of these estimations describes user confidence in the interpolation. While we primarily use this estimated error (and not the estimates themselves) to assess trajectory quality, data assimilation techniques like this have inherent value. For example, Gaillard et al. (2016) and Nielsen-Englyst et al. (2023) used similar techniques to produce gridded datasets of oceanographic data of interest. Schaeffer et al. (2023) also used a similar method to estimate the mean sea surface. Although float data assimilation is outside the scope of this work, we choose float control based on assimilation error to task floats with measuring high-priority undersampled areas.

The contributions of this paper are (1) a comprehensive description of the FloatCast modular float control aid; (2) deterministic and stochastic demonstrations of FloatCast in simulations where it is applied to historical float data from the Gulf Stream; and (3) real-time experimental application of FloatCast to directly control floats in the Philippine Sea, including a component-wise performance assessment. For the field experiment, we used FlowPilot communication mechanisms (Szuts et al., 2023) to send dive commands from FloatCast to the deployed floats. Although ocean dynamics on this scale are stochastic, the results from live FloatCast control are promising.

Section 2 provides a comprehensive description of FloatCast, including necessary background information on ESNs, profiling float characteristics, and the mapping error scoring metric. Section 3 discusses results from two simulation case studies and a field experiment. First, simulation results are presented from case studies using historical float data from a Gulf Stream deployment. Then, experimental results are shown for the period of live FloatCast control of floats in the Philippine Sea. Section 4 summarizes the paper and discusses future work.

## 2 Materials and methods

FloatCast has three modules (Figure 1) that plan float control. These modules are the flow forecaster, the float trajectory integrator, and the dive command selector. The flow forecaster (Module 1) is built around an ESN, a machine-learning method for predicting surface flow fields. The float trajectory integrator (Module 2) uses a simple mathematical model to predict float trajectories. These predictions are based on float locations, dive commands, and the surface flow forecasts from the previous module. The dive command selector (Module 3) assesses the expected quality of various dive commands, ranks the trajectory predictions for each candidate command set via a mapping error metric, and chooses the best one. FloatCast thus combines machine learning, feedback control, and optimization to make informed control decisions for a small fleet of floats. Ideally, these control decisions maximize the floats’ drift using favorable currents. The optimal set of float dive commands is sent to the floats using the FlowPilot software (Szuts et al., 2023). The fundamental mechanisms of the FloatCast software are detailed below.

### 2.1 Flow forecasting module: echo state network prediction tool

The first module of FloatCast forecasts the ocean surface flow field, as shown in Figure 1. We have chosen to predict ocean velocity with an ESN based on satellite altimetry observations. Specifically, we use a product of Sea Level Anomaly (SLA) (E.U. Copernicus Marine Service Information, 2025a; E.U. Copernicus Marine Service Information, 2025b), which describes the instantaneous height difference relative to the time-averaged geoid value.

To first order, the ocean’s surface slopes represent geostrophically-balanced surface currents that are a balance between down-slope pressure gradients and the turning caused by Earth’s rotation (Coriolis force). This balance is well resolved by SLA products that are averaged temporally over 2–3 weeks and spatially over 1° in latitude. This inherent smoothing in SLA has a side benefit of removing higher-frequency and wavenumber signals that are ageostrophic^2^—signals that are too small and too quickly evolving to be useful for advecting floats. More fundamentally, using SLA with ESNs is beneficial because it is an observational product, is straightforward to use and interpret, and is available in real time for use with live floats (E.U. Copernicus Marine Service Information, 2025a).

There are multiple advantages to using the data-driven ESN approach for ocean forecasting. First, ESN hyperparameters do not have to be finely tuned to achieve good performance (Lukoševičius, 2012). This increases FloatCast’s versatility, allowing it to forecast various ocean regions without adjusting hyperparameters like the learning rate, regularization coefficient, reservoir size, or spectral radius. Second, training ESNs is not very computationally demanding, which is helpful because ESNs can be retrained using the latest available ocean data. As a result, predictions from these ESNs are tailored to the specific characteristics of the region at any given time. The ESN training process itself is also expanded to leverage computational savings further. When a new ocean forecast is needed, multiple ESNs are created at once. From this set of ESNs, the forecast from the best-performing ESN is selected for use in the remainder of the FloatCast pipeline. Together, the versatility of applying and training ESNs makes them a suitable choice for the ocean forecasting problem.

#### 2.1.1 Echo state networks: formulation, training, and application

**FIGURE 2:**
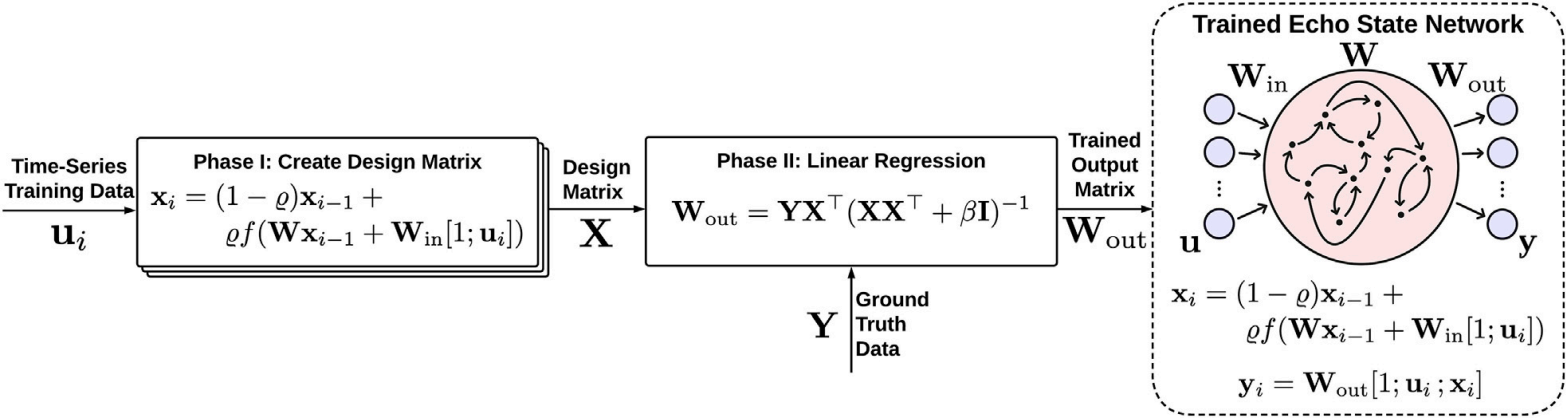
Method for training the echo state network, which FloatCast uses to make Sea Level Anomaly (SLA) predictions. The SLA predictions are later converted to surface flow predictions. The $W_{\text{in}}$ and W matrices are randomly initialized prior to training; ϱ and β are scalars; f is the tanh function. In phase I, the design matrix $X=\left\{\left[1;u_{i}\;;x_{i}\right]\right\}$ is constructed iteratively using input SLA data $u_{i}$ and computed reservoir states $x_{i}$ , for i=1,…,n . In phase II, Tikhonov regularization is used to solve for $W_{\text{out}}$ using the design matrix and ground truth SLA data, $Y=\left\{y_{1},y_{2},\ldots ,\;y_{n}\right\}$ . To make predictions with the trained ESN, the equations in the dashed box are used together. Note that the vector subscript i indicates the time step $t_{i}$ . This graphic is based on information and illustrations in Goswami et al. (2022), Lukoševičius (2012), and Cucchi et al. (2022). Figure modified with permission from its original form in Tolone et al. (2025).

Here, we provide a concise description of the ESN training process based on the notation and methods presented in Lukoševičius (2012). At a basic level, an ESN is a type of recurrent neural network that converts an input time series into a history-dependent representation of its recent dynamics, enabling accurate prediction of future values. The representation is encoded in the input, reservoir, and output weight matrices, which are utilized via algebraic matrix-vector operations paired with specialized information storage. A sketch of ESN training and usage is provided in Figure 2.

The heart of an ESN is the input, reservoir, and output weight matrices, written as $W_{\text{in}}$ , W , and $W_{\text{out}}$ . They have dimensions, $N_{x}\times \left(1+N_{u}\right)$ , $N_{x}\times N_{x}$ , and $N_{y}\times \left(1+N_{u}+N_{x}\right)$ , respectively, where $N_{u}$ is the size of an input, $N_{x}$ is the size of the reservoir, and $N_{y}$ is the size of an output. For our purposes, $N_{u}$ and $N_{y}$ are equivalent and are equal to the number of SLA data points being forecasted. Before training, the $W_{\text{in}}$ and W matrices are randomly initialized following the guidelines of Lukoševičius (2012). The $W_{\text{out}}$ matrix is then trained via the following process.

In the first phase of training, daily input SLA data $u_{i}$ is sequentially fed into

$$x_{i}=\left(1-\varrho \right)x_{i-1}+\varrho f\left({Wx}_{i-1}+W_{\text{in}}\left[1;u_{i}\right]\right),$$ (1)

where each resulting reservoir state vector $x_{i}$ is used recursively in subsequent iterations. The scalar leaking rate ϱ is a tunable hyperparameter; f is the element-wise tanh function. The design matrix, $X=\left\{\left[1;u_{i}\;;x_{i}\right]\right\}$ for i=1,…,n time steps, is constructed from the input data and reservoir state from each iteration of Equation 1. In the first use of Equation 1, $x_{0}$ is the zero vector. The bracket notation used here indicates a column vector.

The second phase of training solves for $W_{\text{out}}$ . Let $Y=\left\{y_{1},y_{2},\ldots ,y_{n}\right\}$ be the known output SLA data from the training dataset, where $u_{i+1}=y_{i}$ , for i=1,…,n . Thus, Y assembles the next time step of SLA data. Given that X is also organized by time step, then for the ideal $W_{\text{out}}$ matrix, the following equation must be true:

$$Y=W_{\text{out}}X.$$ (2)

Using Tikhonov regularization, Equation 2 can then be solved for $W_{\text{out}}$ , i.e.,

$$W_{\text{out}}={YX}^{⊤}{\left({XX}^{⊤}+\beta I\right)}^{-1}.$$ (3)

Here, the scalar β is a tunable hyperparameter and I is an identity matrix.

With $W_{\text{out}}$ trained, ESN predictions are made by chaining together the output of Equation 1 with the vector form of Equation 2, which yields

$$y_{i}=W_{\text{out}}\left[1;u_{i}\;;x_{i}\right].$$ (4)

The process begins by seeding Equation 1 with the newest piece of SLA data $u_{i}$ and the last state from the training stage $x_{i-1}$ to calculate the new state $x_{i}$ . The $u_{i}$ and resulting $x_{i}$ values are then fed into Equation 4, where the resulting $y_{i}$ becomes the SLA forecast for the next day. This process is repeated for as many time steps as desired, with the forecasted $y_{i}$ used as the input $u_{i+1}$ for each subsequent time step. This mathematical formulation is followed for the construction and use of all ESNs in this work.

#### 2.1.2 Additional considerations for FloatCast echo state networks

There are some additional practical considerations for the construction and usage of FloatCast ESNs. One consideration is defining the link between the ESN predictions of surface height, or SLA, and the ocean velocity needed to calculate float advection. Standard oceanographic theory links these by the equation for geostrophic balance $u_{\text{geo}}=-\left(g/f\right)\nabla \times H\hat{z}$ , where $u_{\text{geo}}$ is the geostrophic surface velocity, g is gravity, f is the Coriolis frequency, and H is SLA (Gill, 1982; Choi, 2016).

The forecasting method thus has two steps: (1) forecast the SLA grid for the given region, and (2) convert the SLA forecast to surface flow. This approach halves the computational demand by forecasting scalar heights instead of two-dimensional velocity vectors. As an added benefit, because smooth scalar fields describe a potential function that is non-divergent (Gill, 1982), the scalar prediction satisfies mass conservation without applying additional constraints to the vector fields. To further reduce the ESN’s computational demand, the spatial resolution of the training data is downsampled from a resolution of 0.125° to 0.25° , noting that the original SLA fields contain minimal signal at these small scales.

Although larger ESNs (i.e., larger W matrices) generally outperform smaller ones (Lukoševičius, 2012), increasing the ESN size also quadratically increases the required memory as the matrix dimension increases. Moreover, the spatiotemporal resolution of the surface flow data used here is relatively low, so devoting maximal computational resources to flow prediction is unlikely to generate increased skill. Instead, FloatCast uses an iterative ESN training strategy to improve performance.

The iterative training strategy has two steps. In the first step, a small number of ESNs are trained using the same hyperparameters since performance can vary due to randomness in matrix initialization (Lukoševičius, 2012). These candidate ESNs are ranked according to their prediction error, and the best one is selected for further investigation. In the second step, FloatCast uses the transferable values from the best ESNs from the first step ( $W_{\text{in}}$ and W ), perturbs a hyperparameter of interest, and retrains new ESNs from this foundation. For this work, the retuned hyperparameter is the Tikhonov regularization coefficient β from Equation 3. The best ESN from the second step is used in the remaining modules of FloatCast. This training method increases the memory requirement linearly with the number of ESNs trained rather than quadratically with the matrix size. Moreover, it provides a safeguard against unfavorable random variations in ESN matrix initializations.

**FIGURE 3:**
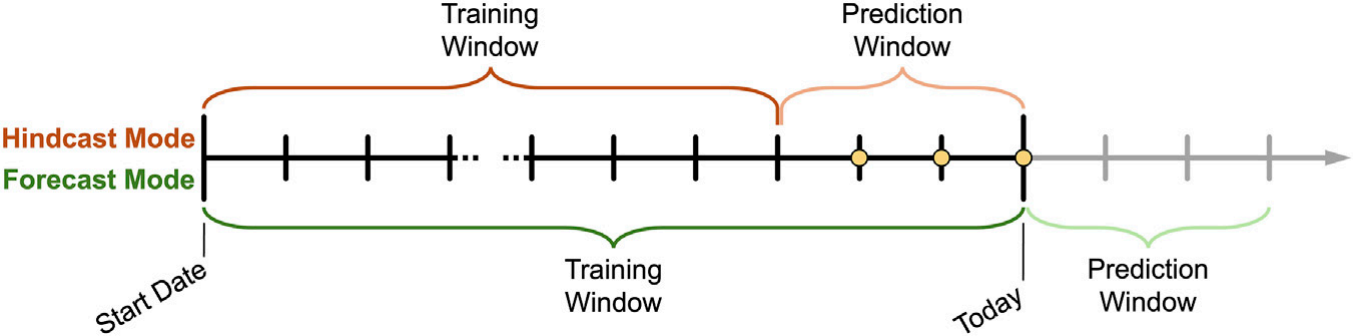
Echo state network timing windows for hindcast mode (top) and forecast mode (bottom). The training data are sketched in black. The hindcast mode training window (dark orange) stops short of covering the entire training dataset available. The hindcast mode prediction window (light orange) extends through the latest piece of training data. The gold dots mark time steps where hindcast predictions can be compared to ground truth data. The forecast mode training window (dark green) covers the entire training dataset. The forecast prediction window (light green) extends into the future, beyond the current date. For the results presented here, both hindcast mode and forecast mode open the training window on 1 January 2000. This ensures that there is enough training data to make sufficiently accurate predictions.

Every FloatCast ESN is generated in both hindcast and forecast mode so that relative ESN performance can be assessed based on its prediction error (Figure 3). In hindcast mode, the most recent training data are withheld so that the SLA prediction can be compared against reference values. In forecast mode, all available training data are used in order to maximize the prediction potential of the ESN. In real-time operations, the performance of ESN forecasts cannot be assessed until the end of the prediction window. ESN quality is assessed based on the root mean square error (RMSE) of the central prediction region, integrated over the prediction time window, as follows:

$$Total\;RMSE=\overset{T}{\underset{j=1}{\sum }}\sqrt{\frac{1}{N}\overset{N}{\underset{i=1}{\sum }}{\left(Y_{i}\left(t_{j}\right)-Y_{i}^{\text{ref}}\left(t_{j}\right)\right)}^{2}},$$ (5)

where T and N are the number of hindcast days and the number of data points inside the central prediction region, respectively. This metric prioritizes accuracy in the central prediction region because it coincides with the sampling region of interest. For example, if the ESNs predict the SLA evolution in a 9°×9° domain, the central prediction region is a 3°×3° box. The i th SLA data point inside this box for hindcast day j is denoted as $Y_{i}\left(t_{j}\right)$ . Its corresponding reference value is denoted as $Y_{i}^{\text{ref}}\left(t_{j}\right)$ . Because FloatCast ESNs predict SLA, the total RMSE has units of meters.

### 2.2 Trajectory integration module: autonomous profiling floats

**FIGURE 4:**
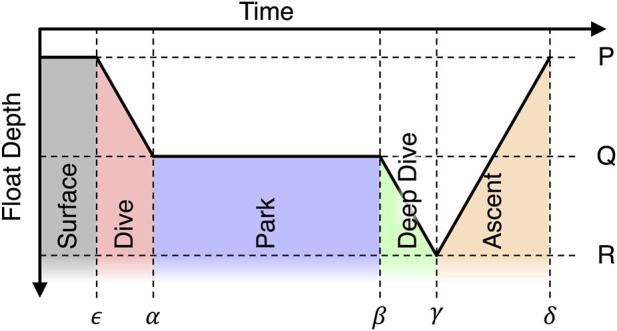
Typical float profiling pattern. The float remains on the surface at depth P until time ϵ to receive new commands. The float then dives to the parking depth Q between times ϵ and α , and remains there for the park duration ( β−α ). The float does a deep dive down to depth R between times β and γ . Finally, the float ascends to the surface from depth R while taking a full profile between times γ and δ . For similar visualizations, see Wong et al. (2020), Wang et al. (2024), and Morris et al. (2024). Figure modified with permission from its original form in Tolone et al. (2025).

We seek to improve the collective sampling performance of a small fleet of profiling floats by modifying their dive pattern characteristics in real time. Profiling floats freely drift with ocean currents and change their vertical depth by adjusting their buoyancy. Thus, our specific objective is to use float depth control to leverage horizontal ocean currents that suit navigational goals. Commercially available profiling floats typically perform the dive pattern sketched in Figure 4. Often, these floats are commanded to repeat a fixed profile, with the canonical example being the Argo sampling pattern of a duration of 10 days, a park depth of 1000m , and a deep-dive depth of 2000m (Wong et al., 2020; Morris et al., 2024). On their ascent to the surface, all floats measure temperature, salinity, and pressure. FloatCast aims to improve sampling effectiveness by actively adjusting the park depth and duration parameters for each float after every dive.

As shown in Figure 4, a float stays at the ocean surface until it receives its park depth and duration commands from FloatCast at time ϵ . Once received, the float begins its initial dive down to the park depth Q . It remains at depth Q between times α and β . Typical park durations ( β−α ) considered here are no longer than 4 days. The float then begins its deep dive to depth R , nominally 2000m . Upon reaching this depth at time γ , the float begins its ascent and takes a full profile along the way. On the surface, floats transmit location and sample data and update their dive commands via satellite communications^3^. This routine repeats until the float battery dies.

#### 2.2.1 Float trajectory prediction model

In order to identify the optimal command set, FloatCast must first generate float trajectories for a variety of candidate commands. Taking the ESN surface flow forecasts, we extrapolate the surface currents downward and apply a Lagrangian particle model to calculate float paths under the different command sets. Subsurface velocities are inferred by multiplying the forecasts of surface geostrophic velocity by an exponential function of depth used previously with success (Szuts et al., 2023). FloatCast thus defines the horizontal float velocity as a function of depth via an exponential decay relative to the surface velocity.

The lateral movement of a float is therefore modeled via the following equations:

$$\dot{x}\left(t\right)=uc\left(a\exp\left[-z\left(t\right)/\zeta \right]+b\right),$$ (6)

$$\dot{y}\left(t\right)=vc\left(a\exp\left[-z\left(t\right)/\zeta \right]+b\right).$$ (7)

At time t , the zonal (positive to the east) velocity of the float is $\dot{x}\left(t\right)$ , and the meridional (positive to the north) velocity is $\dot{y}\left(t\right)$ . The resulting x(t) and y(t) distances are referenced to the origin point of the first dive of the float. These distances are used to obtain updated latitude and longitude coordinates. Float depth at time t is given by z(t) . The nearby zonal and meridional surface flows are written as u and v , respectively. Here, u≜u(x(t),y(t),t) and v≜v(x(t),y(t),t) define the ocean surface velocity closest to the current float location at time t . For a given simulation, FloatCast is designed to seamlessly switch between measured and ESN-predicted values for u and v depending on the intended usage and available data. In Equations 6, 7, constants a=0.11 , b=0.04 , and ζ=500m were chosen based on prior float deployments. The a and ζ parameters shape the modeled velocity profile with depth. The b parameter ensures that the modeled float drift velocity is always non-zero. The c parameter was chosen by trial and error such that a float on the surface moves at 75% of the altimetric surface velocity. Thus, c=0.75/(a+b)=5 .

To model the vertical behavior described in Figure 4 for a given dive, we ascribe the following piece-wise function for vertical velocity:

$$\dot{z}\left(t\right)=\left\{\begin{array}{cc} 0 & if\;t<\epsilon ,\;or\;\alpha \leq t<\beta ,\\ w & if\;\epsilon \leq t<\alpha ,\;or\;\beta \leq t<\gamma ,\\ -w & if\;\gamma \leq t\leq \delta , \end{array}\right.$$ (8)

where w is a constant representative profiling speed, with 0.12m/s used throughout this work. All other variables are as previously defined. Note that depth and vertical velocity are positive downward.

As Equations 6–8 are separable, we use their analytic solutions to directly compute the float trajectories, keeping this step computationally lightweight. Trajectory predictions for different floats and dive commands are calculated independently. Notably, the model assumes that at all depths, floats move in precisely the same direction as the surface flow. This simplification ensures that the state-space representation of the system can be solved analytically. A consequence of this modeling assumption is that the effectiveness of this approach is heavily tied to the spatiotemporal resolution of the surface flow measurement and prediction data. The case studies in Section 3 show that the model achieves reasonable prediction accuracy despite these assumptions.

#### 2.2.2 Float model practical considerations

During real-world deployments, floats must spend time on the surface of the ocean in order to send and receive data via satellites. To minimize this time, FloatCast prepares the next set of dive commands before the floats surface. Since the next surfacing locations of floats are unavailable to FloatCast while the floats are still underwater, dive commands are selected based on where the floats are expected to surface. We call this the lead method for trajectory prediction because the FloatCast optimization runs from one dive cycle ahead in the future. This methodology was used for the real-time application of FloatCast discussed in Section 3.3, and its accuracy is analyzed in Section 3.3.4. We expect that, in most cases, the lead method for float trajectory prediction is sufficient for choosing adequately optimal commands because of the relatively low resolution of surface flow data.

To quantify the prediction error of the state-space model, float trajectory hindcast predictions can be compared with historical float trajectory data. If historical dive commands are unavailable for making hindcast predictions, replacement dive commands can be heuristically approximated based on measured depth-time data. The analysis of the prediction error uses the following definitions:

$${\tilde{x}}_{j}^{i}≜\left\{{\hat{x}}_{j,k}-x_{j,k}\;|\;i-1<t_{j,k}-t_{j,0}<i\right\},$$ (9)

$${\tilde{y}}_{j}^{i}≜\left\{{\hat{y}}_{j,k}-y_{j,k}\;|\;i-1<t_{j,k}-t_{j,0}<i\right\},$$ (10)

$${\tilde{X}}^{i}≜\left\{{\tilde{x}}_{j}^{i}\;|\;j=1,\ldots ,N\right\},$$ (11)

$${\tilde{Y}}^{i}≜\left\{{\tilde{y}}_{j}^{i}\;|\;j=1,\ldots ,N\right\}.$$ (12)

Here, $x_{j,k}$ and $y_{j,k}$ denote the actual eastings and northings with respect to the origin point of the first dive for float j at the end of cycle k . The analogous predictions of eastings and northings are written as ${\hat{x}}_{j,k}$ and ${\hat{y}}_{j,k}$ , respectively. As for the timing, $t_{j,k}$ is the absolute time that float j ends cycle k . The start of the first cycle of float j is written as $t_{j,0}$ . Moreover, ${\tilde{x}}_{j}^{i}$ and ${\tilde{y}}_{j}^{i}$ represent the set of all easting and northing prediction errors for float j that occurred between i−1 and i days of $t_{j,0}$ . The superset of easting and northing errors for all floats that occurred in this time range are written as ${\tilde{X}}^{i}$ and ${\tilde{Y}}^{i}$ , respectively. In Section 3.2, performance statistics related to ${\tilde{X}}^{i}$ and ${\tilde{Y}}^{i}$ are used to inform stochastic float trajectory predictions for a case study that analyzes uncertainty of command set performance.

### 2.3 Command selection module: mapping error scoring metric

For this study, we use a coordinated sampling goal for multiple floats that attempts to resolve a pre-defined region as well as possible. This goal encourages floats to occupy the full region of interest by rewarding trajectories within the desired area, while limiting that reward for samples that are too closely clustered in time or space. This goal is quantified with a mapping error scoring metric used previously for underwater gliders (Leonard et al., 2007; Paley, 2007). The term mapping error refers to user confidence in the scalar field estimate of a scientific quantity from an optimal interpolation method (Leonard et al., 2007; Paley, 2007; Bretherton et al., 1976). We use this confidence metric in isolation—without the scalar field estimate—for analyzing collective sampling performance (Leonard et al., 2007; Paley, 2007). Although it might be possible to achieve the desired float sampling behavior with a multivariable reward function, doing so may also depend on arbitrary weighting constants. The mapping error scoring metric removes this ambiguity by basing its analysis on the spatial and temporal decorrelation scales for the given region.

Mapping error is fundamentally an analysis of the covariance between measurements and a grid of reference points (Leonard et al., 2007; Paley, 2007; Bretherton et al., 1976). The grid of reference points defines the locations where the scalar field predictions and their associated confidence can be calculated based on accumulated measurements. In FloatCast, the reference point grid defines the region of interest that the floats should occupy. The actual covariance between two data points is computed as (Paley, 2007)

$$C\left(R,t,R^{\prime },t^{\prime }\right)≜\sigma_{0}e^{-\frac{\Gamma \left(R,R^{\prime }\right)}{\sigma }-\frac{\left|t-t^{\prime }\right|}{\tau }}.$$ (13)

Here, R and t represent spatial coordinates and time of a data point, respectively. The variance of the scalar measurement field about its mean is $\sigma_{0}$ . The distance function is denoted by Γ . Moreover, σ and τ are the regional spatial and temporal decorrelation scales, respectively. When a pair of data points (R,t) and $\left(R^{\prime },t^{\prime }\right)$ are close to each other in space and time, the resulting covariance is high. Conversely, the covariance is low when this pair of data points is far apart in space or time.

Using Equation 13, the covariance between all measurements is quantified via the measurement covariance matrix. The element in the k th row, j th column of the measurement covariance matrix $\tilde{C}$ is (Paley, 2007)

$${\left[\tilde{C}\right]}_{kj}≜{\tilde{\sigma }}_{0}\delta_{kj}+C\left(R_{k},t_{k},R_{j},t_{j}\right).$$ (14)

The $\tilde{C}$ matrix in Equation 14 is B×B dimensional, where B is the number of measurements. The measurement noise variance constant is denoted by ${\tilde{\sigma }}_{0}$ . Note that $\delta_{kj}$ equals one if k=j , and 0 otherwise.

Using the inverse of the measurement covariance matrix, the covariance of the error for the scalar field estimate $\hat{C}$ is defined by Leonard et al. (2007), Paley (2007), Bretherton et al. (1976)

$$\hat{C}\left(R,t,R^{\prime },t^{\prime }\right)≜C\left(R,t,R^{\prime },t^{\prime }\right)-\overset{B}{\underset{k=1}{\sum }}\overset{B}{\underset{j=1}{\sum }}C\left(R,t,R_{k},t_{k}\right){\left[{\tilde{C}}^{-1}\right]}_{kj}C\left(R_{j},t_{j},R^{\prime },t^{\prime }\right).$$ (15)

This quantity defines confidence in the estimates of the scalar field that would be produced by these measurements (Bretherton et al., 1976). Lower values for Equation 15 indicate lower error and better performance.

Integrating the covariance of the error (Equation 15) over the region of interest B , we formulate the scoring metric ψ(t) used to evaluate candidate trajectories as (Leonard et al., 2007; Paley, 2007)

$$\psi \left(t\right)≜-\log_{10}\left(\frac{1}{\sigma_{0}|B|}\int_{B}\hat{C}\left(R,t,R,t\right)dR\right).$$ (16)

The desired outcome is to minimize this area integral of the error covariance. The negative logarithm inverts this integral such that ψ(t) increases as the error decreases. We seek to maximize the discrete-time integral of Equation 16 in the given region and time window of interest, as determined by the user. The performance scores of all permutations of dive commands are calculated based on the expected performance of the entire fleet of floats. FloatCast selects the optimal set of fleet-wide commands to transmit to floats based on the overall performance score rankings.

By optimizing commands for the whole fleet, FloatCast accounts for how dive commands for neighboring floats affect each other. This encourages the selection of complementary commands across floats. Although outside the scope of this paper, a sensitivity analysis could provide insight into how this method of fleet-wide analysis affects the commands chosen for different fleet configurations. Some characteristics to consider are the number and spacing of nearby floats, the stagger in float surfacing times, and the regional spatial and temporal decorrelation scales ( σ and τ , respectively; see Equation 13).

## 3 Results

To demonstrate FloatCast capabilities, we present two simulation case studies and a real-world implementation. The first simulation case study uses deterministic float-trajectory predictions that inherently assume the float motion model perfectly captures float dynamics (Section 3.1). The second simulation case study uses stochastic float trajectory predictions that incorporate uncertainty into the float command selection process (Section 3.2). Both of these case studies are based on historical data from a float deployment in the Gulf Stream during July 2024. Float trajectory data from this float deployment have previously appeared in Tolone et al. (2025). Following this, experimental results from live FloatCast control of floats in the Philippine Sea during November–December 2025 are presented (Section 3.3). Control decisions for this deployment were made based on deterministic float trajectory predictions.

### 3.1 Deterministic simulation case study using historical Gulf Stream float data

This case study demonstrates how FloatCast selects optimal dive commands for a given configuration of floats using a deterministic float model. We walk through one iteration of the FloatCast method to show how each step is implemented. We examine three floats that were deployed in the North Atlantic in the Gulf Stream near the New England Seamount region. The strong Gulf Stream currents are expected to give floats more control authority, but reduce their ability to stop undesirable drift from unfavorable currents. For this system configuration, the FloatCast optimization revealed that sampling frequency is more important than float spacing in selecting dive commands that best resolve the region of interest.

#### 3.1.1 Deterministic simulation setup

For this case study, FloatCast was programmed to have historical knowledge of float and ocean data from the Gulf Stream deployment for the week leading up to noon on 17 July 2024. Starting there, FloatCast is tasked with finding the fleet-wide command set that maximizes the scoring metric (Equation 16) over the region of interest spanning 37°N to 40°N and 65°W to 62°W . Candidate ESNs are constructed to predict SLA in the larger domain from 34°N to 43°N and 68°W to 59°W . In the ESN construction process, relative performance is assessed by evaluating the accuracy of hindcast SLA predictions in the smaller 3°×3° region of interest. The ESN is trained in forecast mode using historical SLA data from 1 January 2000 through 17 July 2024. The first day of the ESN forecast is 18 July 2024.

This case study evaluates dive commands for three floats. Float trajectory predictions are made deterministically and originate from the last known surfacing locations of each float prior to noon on 17 July 2024. Since this analysis is performed retroactively with all surface positions already known, there is no need to implement the lead method for float trajectory prediction introduced in Section 2.2.2. In the simulation, FloatCast predicts float trajectories 10 days into the future, with the same float command used throughout this duration.

**FIGURE 5:**
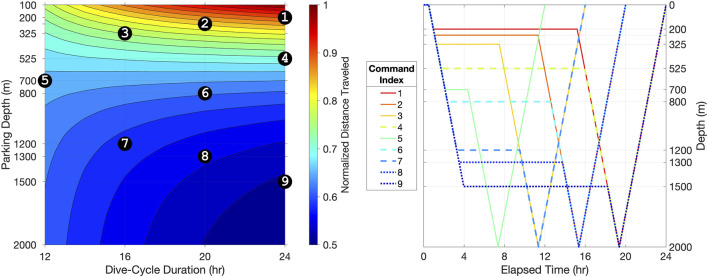
(Left) Normalized distance traveled in a uniform flow field under a variety of different command combinations. The expected distance traveled for a command set was calculated over 100 days using a fixed dive velocity w=0.12m/s and uniform flow field (u,v)=(1,0)m/s . The surface time between dives is assumed to be 30 min to allow for float communications. The deep dive depth is fixed at 2000m for each command so that floats take a full profile on their ascent to the surface. Distances are normalized by the maximum value calculated. To maximize the expected variety of prediction outcomes, nine dive commands were chosen by inspection that represent diverse scores from the normalized distance traveled metric. The dive commands selected are marked with black dots. Only four dive durations are considered for simplicity. (Right) Profiling patterns for the selected commands. Portions of this figure were adapted from Tolone et al. (2025) with permission.

Candidate commands must be selected judiciously, as computation time increases exponentially with the number of floats. Here, we seek to maximize the expected variety in float trajectories from candidate command sets using the following selection process. Starting with a simple flow field (u,v)=(1,0)m/s that is uniform and constant across time and space, we integrate Equation 6 to solve for the position of the float 100 days into the future, assuming a constantly repeating dive command. We consider dive durations spaced hourly from 12 to 24 h, and parking depths spaced by 25m from 100 to 2000m . The results of the integrations are normalized by the maximum distance traveled by any command set (in this case, 24 h at 100m park depth), as depicted in Figure 5 (left).

From the batch of commands shown in Figure 5 (left), nine are chosen that have diverse normalized distance scores and span the depth range. The profiles from these commands are shown in Figure 5 (right). These candidate commands provide several distance options while preserving access to depth-varying flow direction. In a single iteration of FloatCast, each float can choose a different dive command. Since there are nine commands and three floats, there are $9^{3}=729$ possible command set combinations to be assessed. For this case study, it is feasible to exhaustively test all of these command set combinations with reasonable computation effort and time.

Trajectory predictions in FloatCast are evaluated using the mapping error scoring metric parameterized as follows. A reference grid is made within the 3°×3° region of interest so that each reference point is at the center of a 0.5°×0.5° area. Spatial and temporal decorrelation scales are set to 25km and 1.25days , which were chosen as the smallest scales at which geostrophic balance applies at this latitude. The variance of the scalar measurement field about its mean $\sigma_{0}$ was set to 1, and measurement noise variance ${\tilde{\sigma }}_{0}$ was set to 0.1. Measurements older than 3 temporal decorrelation scales are excluded from the mapping error calculation because they have minimal impact. The mapping error calculations are referenced to midnight each day from 18–28 July 2024.

#### 3.1.2 Deterministic simulation results

**FIGURE 6:**
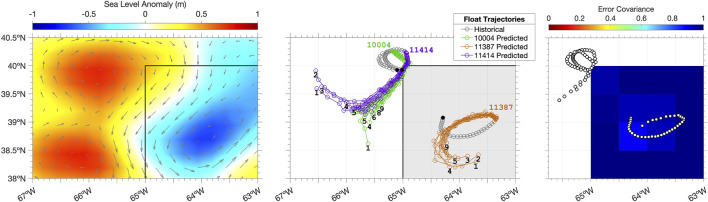
FloatCast outputs for a single iteration retroactively applied to the Gulf Stream deployment. (Left) ESN forecast snapshot from 24 July 2024 in the region where these floats were active. Note the dominant influence of the large eddy-like features. (Middle) Historical float trajectories leading up to the start of the prediction window are shown in gray, with black dots marking the starting locations. Predicted float trajectories for candidate commands are shown in green, orange, and purple for the prediction window, which is from about noon on 17 July 2024 to about noon on 27 July 2024. Select trajectories are labeled with the command index used to generate them based on the numbering scheme in Figure 5. The best-performing command set was for all three floats to use command index 5. (Right) Error covariance snapshot from 24 July 2024 for the float trajectory predictions of the selected command set combination. The error covariance is only calculated inside the region of interest, which spans 37°N to 40°N and 65°W to 62°W . The plot window is shifted from the region of interest to show the float trajectories at this point in time.

The outputs of FloatCast for a single iteration under the parameterization described above are given in Figure 6. These plots show how each FloatCast module contributes to the dive command optimization. In this particular example, the given system configuration and spatial and temporal decorrelation scales meant that inter-float spacing was less important than maximizing sampling frequency.

A snapshot of the ESN forecast on 24 July 2024 is shown in Figure 6 (left), centered around the area of expected float traversal. The ESN predicts three eddy-like features in this area, which greatly influence the predicted motion of the floats. These features are the primary mechanism of lateral control for the floats and can either keep them close to the region of interest or carry them away. Trajectories integrated in this flow field are sensitive to initial conditions and control plans applied.

The complete set of possible float trajectories predicted by the Lagrangian particle model is shown in Figure 6 (middle). As expected, float trajectories for shallower park depths tend to cover more distance than those that use deeper park depths. Even though floats 10004 and 11414 are moving through similar regions of the ocean, their trajectories diverge under command set 1, which is a day-long shallow dive. This illustrates a point in space and time where a bifurcation in the flow field has occurred. Such features can be used advantageously for navigation, even though this specific feature appears to be only minimally helpful for this case study. This is because these two floats are expected to be swept away from the region of interest regardless of the commands used. From the set of all predicted float trajectories, the mapping error performance score is computed for each combination. As noted above, there are $9^{3}=729$ possible command combinations to choose from because there are nine command sets and three floats. Floats followed the same command throughout the prediction window to reduce the number of command set permutations.

Figure 6 (right) contains a snapshot of the error covariance $\hat{C}$ for the best-performing command set on 24 July 2024 projected onto the region of interest. The color of each pixel in the region of interest indicates the freshness and proximity of the collection of float samples to the reference point at each pixel’s center. Lower values for error covariance are more desirable than higher ones because the performance metric in Equation 16 takes the negative logarithm of the area integral of the error covariance. This translates lower error-covariance values into higher performance scores. In this case, the best option was for all floats to follow the rapid half-day dives, which are denoted with an index of 5 in Figure 6 (middle). This command set is not expected to separate floats 10004 and 11414 from each other, but it increases the sampling rate for each float. This shows that sampling frequency is of greater value than float separation for dive command selection in this case study. This is likely due to fast flow fields and the relatively short spatial and temporal decorrelation scales used here in the mapping error scoring metric.

### 3.2 Stochastic simulation case study using historical Gulf Stream float data

Using the same system configuration as the simulations in Section 3.1, this case study examines how FloatCast can incorporate uncertainty in its control decisions. This is primarily done by introducing stochasticity into float trajectory predictions using Monte Carlo trials. To mitigate the computational demand of the stochastic approach, the number of performance score calculations is limited via the two-stage greedy search method discussed below. In stage I of this method, the performance of trajectory predictions for a random subset of command sets is evaluated, and the best ones are set aside. Stage II evaluates another batch of predictions for command sets that are similar to the best commands from stage I of the analysis. The highest-ranking command set from this pool of evaluations is then selected for use. The method for choosing command sets in stage II is based on the expected distance traveled metric introduced in Section 3.1.1 (Figure 5). The results here indicate that this greedy search method of selecting dive commands is promising.

#### 3.2.1 Stochastic simulation setup

This case study considers the same mission scenario from Section 3.1. FloatCast is again tasked with selecting the next set of dive commands for the three floats to use for the current time step, which starts at about noon on 17 July 2024. The same ESN forecast data, candidate dive commands, and mapping error parameterization from the previous case study are used again for this example. However, FloatCast now considers prediction uncertainty in the analysis.

FloatCast incorporates uncertainty in the predictions and evaluations of each command set by using stochastic Monte Carlo trials. This is done by expanding the float trajectory prediction step so that every command set is tested multiple times, with randomness injected into each prediction. These repeated stochastic predictions for a given command set are its Monte Carlo trials. In each trial, predicted float surfacing locations are perturbed using a random normal distribution. This sets the new surfacing locations that seed the remaining dive predictions in the given simulation. The mean of the normal distribution used is 0km/day and the standard deviation scales with expected prediction uncertainty.

**FIGURE 7:**
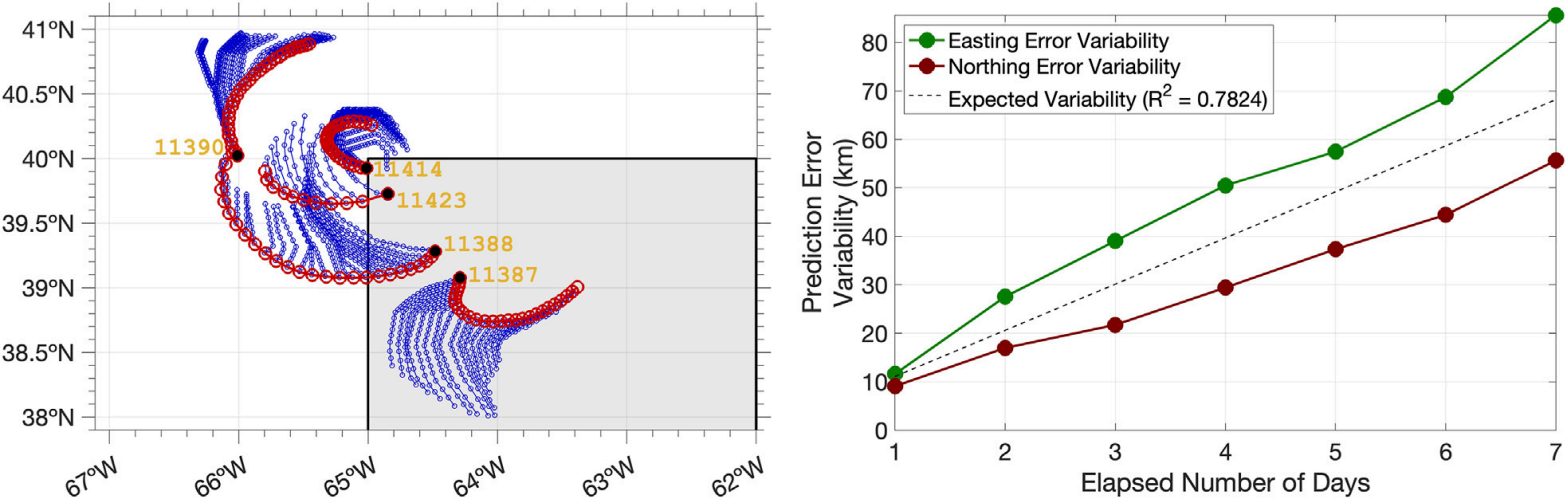
(Left) Hindcast float trajectory predictions (blue) originating from the historical surfacing locations (red) of five out of the ten floats considered in this analysis. Each prediction extends through the current time step and uses dive commands determined heuristically from the historical float depth data. The starting point of the historical trajectories is marked with a black dot. Prediction accuracy varies. (Right) Prediction error variability growth rate for the float trajectory hindcasts in this simulation. The standard deviation of the easting ${\tilde{X}}^{i}$ and northing ${\tilde{Y}}^{i}$ prediction errors are shown here referenced to the elapsed number of days i at the close of each timing interval. The error variability growth rate is 9.5173km/day , as determined by the slope of the dashed line from the linear regression.

For Monte Carlo trials, the standard deviation of the normal distribution used to perturb dive end points is determined based on the accuracy of float trajectory predictions leading up to the current time step. To evaluate the recent prediction accuracy, we construct and compare float trajectory hindcasts for ten floats starting from 10 July 2024 through the current time step of noon on 17 July 2024. Starting from each surfacing location in the historical float trajectories, predictions are made up to the current time step using dive commands determined heuristically from the historical float depth data. The resulting trajectory predictions originating from five of the ten historical float trajectories are shown in blue in Figure 7 (left). As this figure illustrates, the veracity of FloatCast predictions varied. The hindcast flow field from the ESN used in Section 3.1 informs these float trajectory predictions. The prediction accuracy is now quantified in order to set the standard deviation of the normal distribution.

For each prediction made in this analysis, the northing and easting errors are computed by subtracting the actual eastings and northings from the predicted values, as defined by Equations 9, 10. Prediction errors are grouped based on the elapsed number of days from the start of the prediction, as shown by Equations 11, 12. The standard deviation of the set of prediction errors for each elapsed time interval (i.e., ${\tilde{X}}^{i}$ and ${\tilde{Y}}^{i}$ for i=1,2,… ) is computed and plotted in Figure 7 (right). As this plot shows, there is approximately a 10km/day error variability growth rate. This rate is used as the standard deviation of the normal distribution for perturbing dive end points in the stochastic Monte Carlo trials. Thus, when a float surfaces in a Monte Carlo trial integration, the dive end point is perturbed northward and eastward by distances given by multiplying the dive-cycle duration by a rate value taken from this random normal distribution. Recall that the mean of this normal distribution is 0km/day .

In the stochastic analysis part of this case study, 100 Monte Carlo trial predictions are made for floats following each candidate dive command. These predictions are evaluated by analyzing the collective behavior of the Monte Carlo trials for the group of floats under different combinations of command sets. In this case, a command set consists of the specific dive commands given to all three floats in the simulation. The performance scores from each batch of Monte Carlo trials for a given command set form a distribution characterizing expected performance. Command sets are chosen based on the average performance score across their Monte Carlo trials. The uncertainty of performance for a command set is measured by the standard deviation of the performance scores from its Monte Carlo trials.

Since there are nine commands for the three floats to choose from, there are again 729 possible command set combinations. With the Monte Carlo trials, this means that there are 72,900 results for which performance scores can be computed. Although not technically prohibitive here, it is clear how this computation could become impractical as fleet size and the number of candidate commands increase. The two-stage search method discussed below significantly reduces the computational load by limiting the number of command sets tested.

During stage I, the performance scores of 100 command sets are computed, and the three best are selected for further analysis. In stage II, performance scores are computed for command sets similar to the three selected from stage I. The method of determining command sets similar to a reference set is as follows. For each of the selected float commands in the reference set, two commands are chosen that are most similar to it based on the expected distance traveled metric introduced in Section 3.1.1 (Figure 5). For each float, these three commands (the reference command plus the two most similar ones) are saved, leaving three collections composed of three float commands each. Using all possible permutations from these collections, $3^{3}=27$ candidate command sets are then constructed and stored. This is done for each of the three reference sets. If there are no duplicates, there are a maximum of (27)(3)=81 possible command sets to be evaluated in stage II.

For the example presented in Section 3.2.2, several duplicates had to be removed so that only 54 command sets were tested in stage II. In this case, the two-stage search method therefore reduced the number of performance score calculations from 72,900 to 15,400. For this example, this method is able to maintain sufficient optimality despite this reduction in the search space—albeit aided by random chance. For active float control, the tradeoff between optimality and computation time is only necessary when an exhaustive search is not feasible in real time.

#### 3.2.2 Stochastic simulation results

**FIGURE 8:**
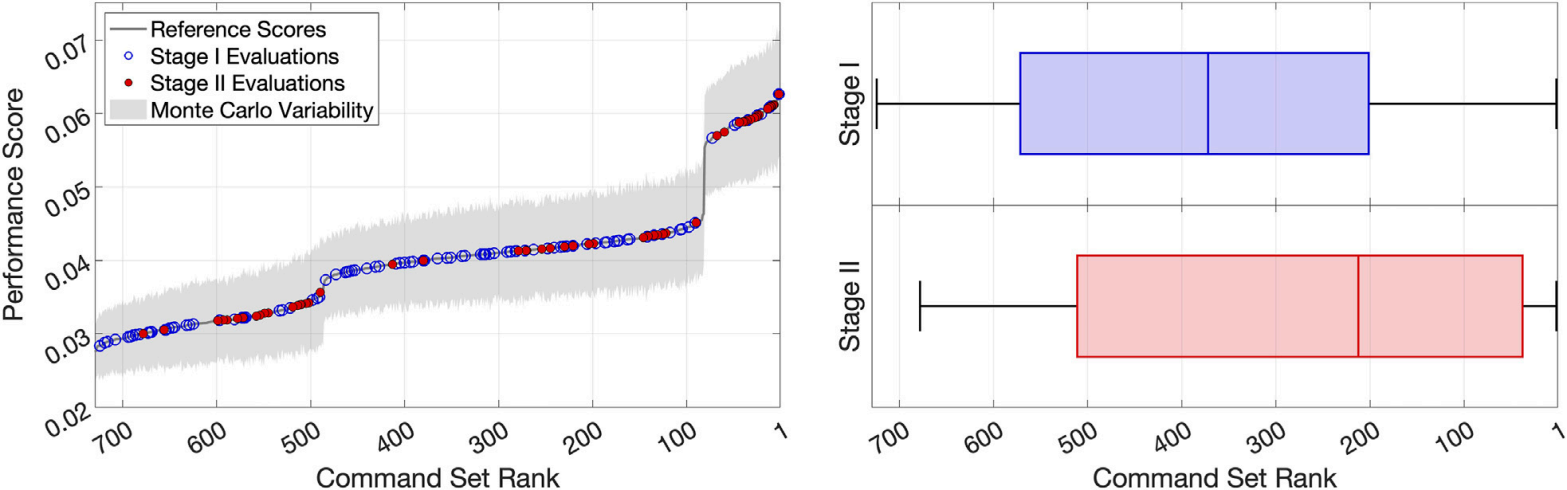
These plots organize command set combinations in terms of their relative ranking against one another along the horizontal axis. (Left) Monte Carlo trial average performance scores (gray line) plus/minus one standard deviation (gray shading) plotted against command set ranks. The ranks of command sets evaluated during stages I and II are marked with blue circles and red dots, respectively. (Right) Distribution of stage I (blue) and stage II (red) command set rankings. Stage I evaluated 100 command sets, and stage II evaluated 54 command sets. Stage II has a greater concentration of high-performing command sets evaluated, as shown by the upper quartiles of each plot. The upper quartile of stage I is 201.5, and the upper quartile of stage II is 38.

Under the parameterization outlined above, stochastic FloatCast analysis was performed for this system. FloatCast generated sets of float trajectory predictions using Monte Carlo trials. The performance score distributions of select command set combinations were then computed via the two-stage optimization strategy. Figure 8 compares the two-stage optimization results against the reference set of performance score distributions from a complete exhaustive search. The plot of performance scores versus command set ranks in Figure 8 (left) simply shows the performance score averages for each batch of Monte Carlo trials, plus/minus one standard deviation. This plot shows that the design space has uneven features. Moreover, the command set chosen should have a measurable impact because the differences between the best and worst average performance scores exceed the uncertainty bounds.

As this figure shows, the two-stage optimization found a near-optimal solution with far fewer evaluations than the full exhaustive search. Only 15,400 performance score evaluations were needed—10,000 for stage I, and 5,400 for stage II. This is about 22% of the 72,900 evaluations needed for a full exhaustive search. Despite the reduction in the number of command sets analyzed between stages I and II, FloatCast still evaluated a substantial number of near-optimal command sets with the two-stage search. This is seen by examining the distributions of evaluated command set ranks in the box and whisker plots in Figure 8 (right). The upper quartiles for stages I and II are 201.5 and 38, respectively. This shows how stage II evaluated a greater concentration of high-ranking command sets compared to stage I. In the end, FloatCast selected the second-best command set combination. FloatCast was aided by random chance in this example because this command set was initially tested in stage I of the optimization. However, this analysis shows how stage II of the optimization can potentially help identify and test other high-ranking commands.

**FIGURE 9:**
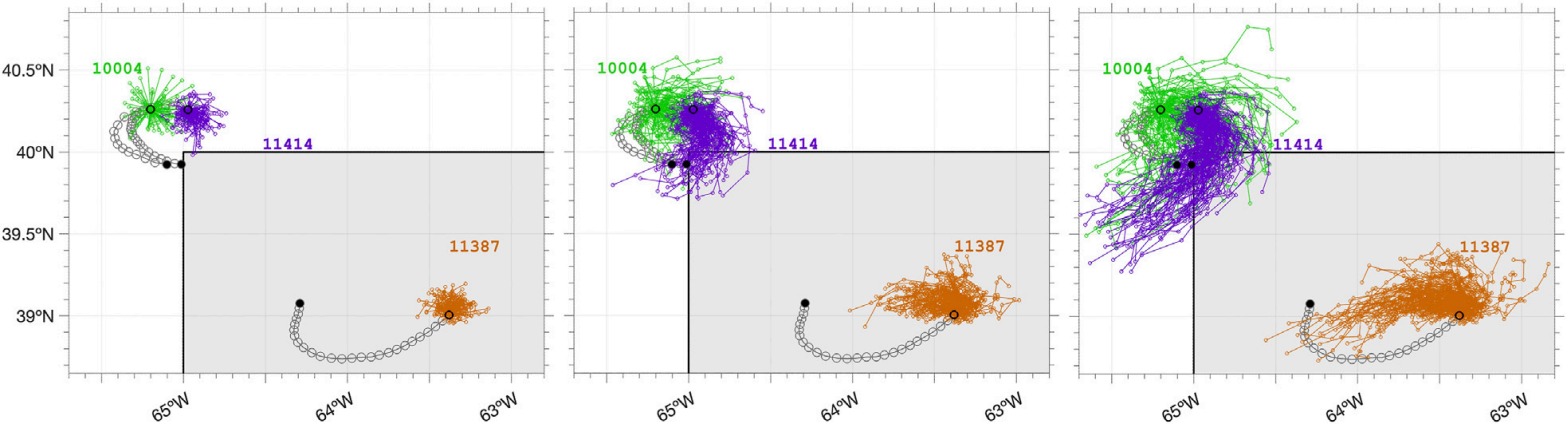
Stochastic Monte Carlo trajectory predictions for the command set chosen by the two-stage optimization. Starting from the black dots, the gray trajectories mark the historical float locations leading up to the prediction window, similar to Figure 6. From left to right, Monte Carlo predictions for each float are shown after 1, 3, and 5 elapsed days from each float’s last surfacing time. The region of uncertainty grows rather quickly, which highlights the need for future work to improve overall prediction accuracy. Note that there are 100 Monte Carlo trials for each float.

The selected command set called for float 10004 to use command index 8 and floats 11387 and 11414 to use command index 5 (Figure 5). Figure 9 shows all predicted float trajectories for the Monte Carlo trials of this command set. As this figure shows, the uncertainty in float trajectory predictions grows rather quickly. The true optimal command set from the exhaustive search of the Monte Carlo trials was for all floats to use command index 5. Although this command set is the same as that found in Section 3.1, the stochastic analysis illustrates prediction uncertainty. Future work could aim to reduce the uncertainty in each prediction. The rapid growth of prediction uncertainty (Figure 9) shows why it is important to frequently make new predictions when FloatCast is used in real time.

### 3.3 Real-time implementation of FloatCast in the Philippine Sea

FloatCast control was implemented in real-time during a float deployment in the West Pacific in the Philippine Sea in November–December 2025. Multiple mission constraints affected the parameterization of FloatCast, the most important of which are specified in Section 3.3.1. Experimental float trajectory data from the FloatCast control window are presented in Section 3.3.2. In order to better understand the performance of FloatCast, the accuracy of its main prediction components is assessed. The error of the ESN forecasts from this deployment is examined in Section 3.3.3. The fidelity of the lead method for float trajectory prediction is discussed in Section 3.3.4. Despite challenges typical of ocean sampling missions, FloatCast successfully performed closed-loop control of profiling floats. Generally speaking, the performance of each FloatCast module during this deployment was promising.

#### 3.3.1 Philippine Sea deployment overview

**FIGURE 10:**
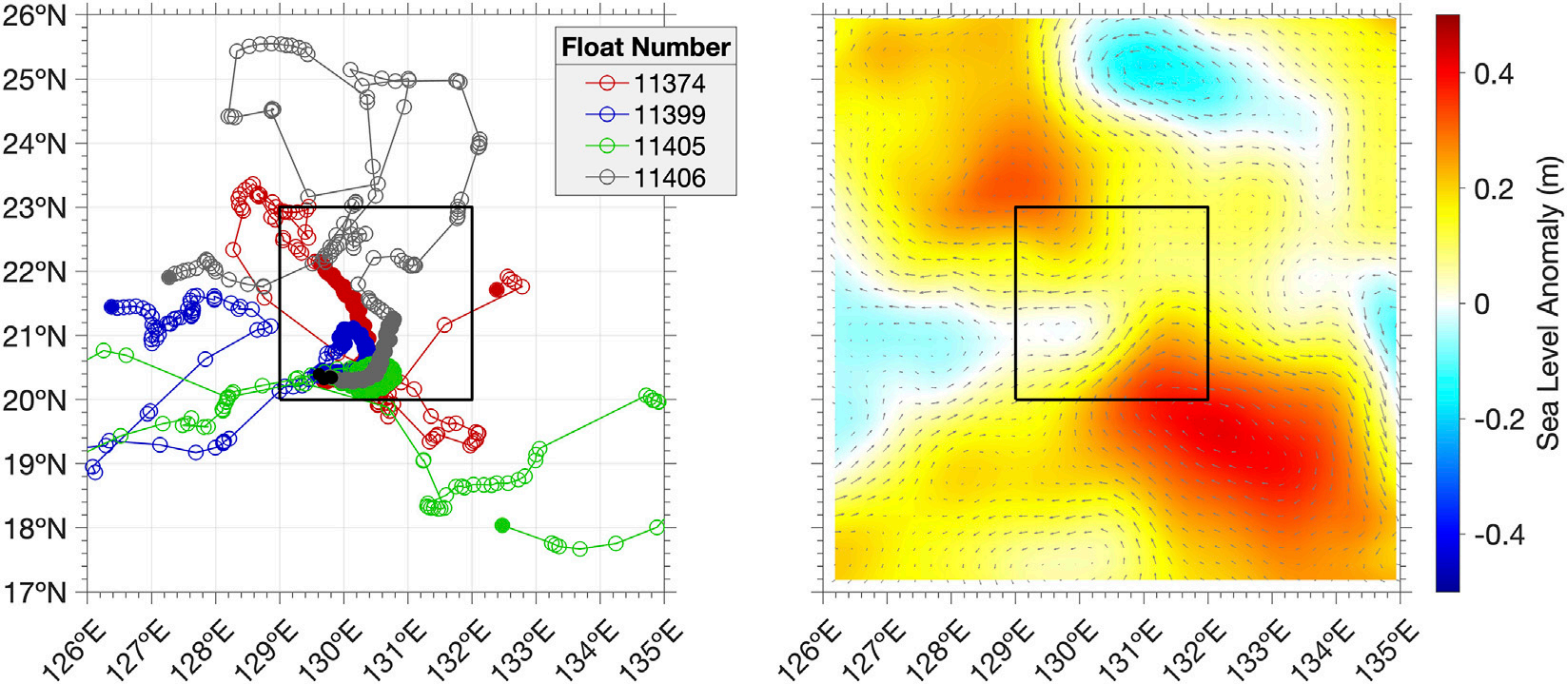
(Left) Overall trajectories of floats from early February 2025 through 29 December 2025. Float starting locations are marked with black dots. Other points indicate float GPS fixes throughout the mission. In the beginning of the deployment, floats performed rapid dives for about 1 month. Imperfect data streams may contribute to some of the large gaps in float trajectories shown above. (Right) Ocean surface dynamics in the Philippine Sea on 13 December 2025. The black box in the middle indicates the region of interest. The arrows represent the surface velocity. The anticyclonic eddy in the bottom right corner of the domain persisted throughout much of the control window and was a key feature of interest.

This field experiment officially began in early 2025 with the deployment of 35 floats in the Philippine Sea near Guam. FloatCast took over crucial aspects of float navigation for the control window from 27 November 2025 to 29 December 2025. FloatCast published daily updated float commands, and FlowPilot mechanisms (Szuts et al., 2023) sent these commands to the floats. While the ultimate goal is for FloatCast to be fully automated, an operator initialized and ran components of the software each day for this field experiment. FloatCast control began when eight floats remained. Over the course of the FloatCast control window, multiple floats failed and went offline due to expected attrition given the demands placed on them over their lifespan. Two other floats were almost never inside the control domain during this period. As a result, there are four float trajectories that will be examined here. The full trajectories for these floats are shown in Figure 10 (left).

FloatCast was tasked with directing floats to take measurements that best resolve the region of interest, which was defined as the box from 20°N to 23°N and 129°E to 132°E . The ESNs made forecasts of SLA in the domain defined from 17°N to 26°N and 126°E to 135°E . The same ESN hyperparameters used to generate the ESN for the examples in Sections 3.1 and 3.2 were used again for this real-time control period. ESN performance was assessed in real time based on hindcast accuracy in the central portion of the prediction domain. The ESN prediction domain was intentionally chosen so that this performance assessment region coincided with the measurement region of interest. Figure 10 (right) illustrates this region partitioning and shows a snapshot of the ocean surface flow dynamics from the control window.

Unlike the example simulations presented in Sections 3.1 and 3.2, FloatCast only chose between two command options for each float. Recall from Section 2.2 that floats are given park depth Q and park duration (β−α) commands. The candidate park depths here were 200 or 1500m . To prolong float battery life, all park durations were restricted so that the total expected dive duration was about 4 days. The deep dive depth R for each dive was fixed at 2000m so that floats would take a full profile on every ascent to the surface. This parameterization makes it possible to compare directly the effect of the shallow and deep park depths on predicted float trajectories. The expectation is that when a float is in a favorable current, it should do a shallow dive to maximize drift. Conversely, if the float is in an unfavorable current, it should do a deep dive to minimize drift. In this context, float depth control essentially becomes a velocity controller with heading angle determined by the environment.

To define the reference point grid for the mapping error scoring metric, the region of interest (the box from 20°N to 23°N and 129°E to 132°E ) was divided into a 6×6 uniform grid of reference points. This is similar to the examples presented in Sections 3.1 and 3.2. Again, each reference point effectively covers a 0.5°×0.5° area, though these are not exclusive borders. In each FloatCast prediction, the mapping error was computed at midnight of each day, starting from the current day and extending 10 days into the future. As before, these reference points were compared against sample points defined by float surfacing locations and times. Like the examples in Sections 3.1 and 3.2, measurement points older than three temporal decorrelation scales τ were removed because they would have minimal impact on the calculation. The spatial and temporal decorrelation scales were increased from their previous values in Sections 3.1 and 3.2 because this region is more homogeneous and stagnant compared to the Gulf Stream. The spatial decorrelation scale σ was 55km , which is an approximation of the Rossby radius for this area (Chelton et al., 1998). The temporal decorrelation scale τ of 1.4583 days roughly matched the inertial period. The variance of the scalar measurement field about its mean $\sigma_{0}$ was not changed from the Gulf Stream example, and was set to 1. The measurement noise variance ${\tilde{\sigma }}_{0}$ was similarly kept at the original value of 0.1.

#### 3.3.2 Real-time FloatCast implementation results

**FIGURE 11:**
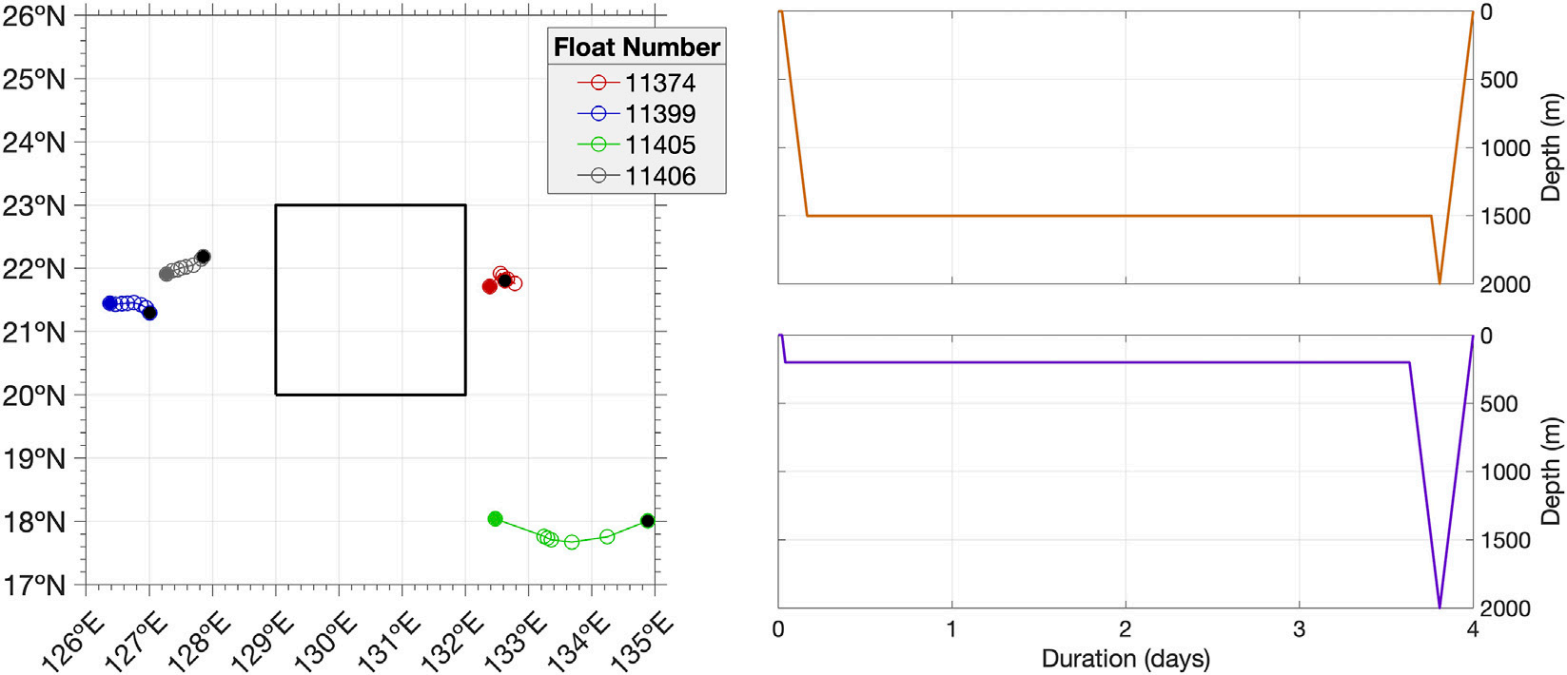
(Left) Trajectories of the four floats of interest during the FloatCast control window. The black dots indicate the starting locations of the floats. Other points indicate float GPS fixes throughout the control window. FloatCast simulations resulted in a mix of shallow and deep dive commands for float 11374. For floats 11399 and 11406, FloatCast consistently chose deep dive commands, likely limiting unfavorable westward drift, as desired. Float 11405 moved much closer to the box than the other floats despite going offline around halfway through the FloatCast control window. This is likely because float 11405 was consistently given shallow dive commands and was located near strong surface currents directed toward the box. (Right) Theoretical deep and shallow profile patterns for FloatCast to choose from. The extended dive duration significantly alters the profile shape relative to those used in Sections 3.1 and 3.2.

For this mission, our objective was to establish closed-loop control of multiple floats using FloatCast and later assess system functionality. The specific role of FloatCast was to publish daily updated float commands that were to be sent to the floats when they surfaced. Dive commands were computed daily to match the update frequency of the SLA data. Throughout the duration of the mission, FloatCast also published the daily ocean surface flow forecasts it used to make the float trajectory predictions. Figure 11 (left) shows the trajectories of four floats over the duration of the FloatCast control window, and (right) shows the depth profiles considered for each dive. Although the floats did not enter the region of interest sketched in Figure 11 (left), the dive commands were chosen so that floats would, in theory, move closer to the box. This was because the scoring criteria valued float trajectories close to the region of interest, even if they were not inside it.

Even though float 11405 went offline around halfway through the FloatCast control window, it traveled farther than the other three floats highlighted in Figure 11 (left). This is likely because float 11405 was in a persistent and strong anticyclonic eddy located near the southeastern corner of the domain, as shown in Figure 10 (right). The clockwise rotation of this eddy moved float 11405 closer to the region of interest. Moreover, FloatCast consistently chose shallow dive commands for float 11405, which might have helped it take advantage of this favorable drift. The westward and slightly northward movement of float 11405 toward the region of interest supports this choice of shallow dive commands. We expect that, if float 11405 had been given deep dive commands, it would still have moved closer to the region of interest, but at a slower rate. Note that there was an instance where float 11405 experienced a minor glitch, causing it to unexpectedly perform consecutive hours-long dives before it returned to normal diving behavior.

The consistent choice of deep dive commands for floats 11399 and 11406 throughout the FloatCast control window is also encouraging. The location data shows that these floats were trapped in a predominantly westward flow that took them away from the region of interest. We expect that the deep dives helped keep these floats closer to the region of interest than shallow dives. FloatCast output mostly deep dive commands for float 11374 throughout the control window. This float did not encounter strong, distinct flow features, which may have contributed to its limited translational movement. There were multiple cases where float 11374 failed to post high-quality GPS data when it surfaced, making it harder to track. This stretched the exception-handling ability of FloatCast, and future work could improve this capability further.

FloatCast optimization was performed for all floats as a group so that command decisions for each float could be theoretically impacted by the others. Alternatively, each float could be optimized in isolation from the others. At this stage, it is not clear to what extent the group optimization approach changed the output commands compared to single-float optimization. However, it is our expectation that group optimization commands are generally superior to single-float optimization commands because of the ability to plan at the level of the fleet.

#### 3.3.3 Daily echo state network performance

This section presents time-series data related to ESN performance in the FloatCast control window. Recall from Section 2.1.2 that FloatCast ESNs are trained to make SLA hindcasts and forecasts. The only difference between these modes is the training and prediction windows used (Figure 3). During the field experiment, the quality of the ESNs used in hindcast mode was assessed based on the total RMSE metric given by Equation 5. Based on the results, FloatCast then selected the ESN forecast to use for the float trajectory prediction step. Now that data for the forecast time windows are available, the performance of the ESN forecasts is also evaluated.

**FIGURE 12:**
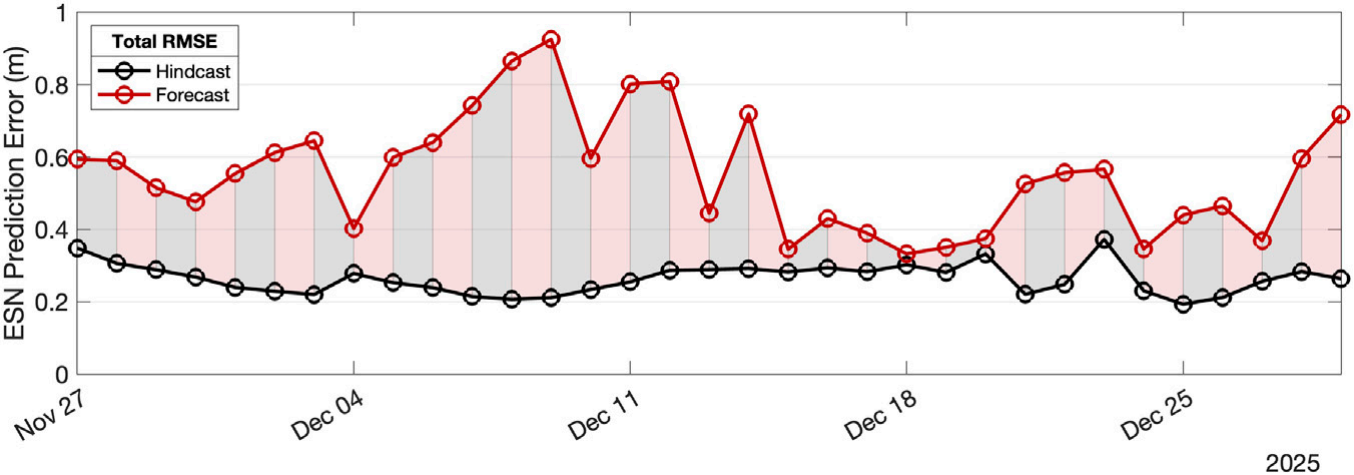
Daily prediction error for the selected echo state networks (ESNs) used in hindcast mode and forecast mode from 27 November end of December 2025. The ESN prediction error is the total root mean square error (RMSE) of the Sea Level Anomaly prediction over the duration of the hindcast/forecast window. The forecast error (red) was typically much higher than the hindcast error (black). The hindcast prediction error had a mean of 0.264m , and a standard deviation of 0.042m . The forecast prediction error was higher and had greater variability, with mean and standard deviation of 0.556m and 0.161m , respectively. In total, 33 echo state networks are analyzed here.

Figure 12 presents the total RMSE time-series data for ESN hindcasts and forecasts. ESN hindcast error values were computed in real time; ESN forecast error values were computed in later analysis. Recall that all FloatCast ESNs predict SLA time-series data. As the plot in Figure 12 shows, the total RMSE for the ESN forecast was typically much larger than that for the ESN hindcast. The mean and standard deviation of the total RMSE time-series data for the ESN forecasts ( 0.556±0.161m ) were roughly two and four times as large as that for the ESN hindcasts ( 0.264±0.042m ), respectively. We believe this is a consequence of the altimeter data reprocessing window overlapping with the ESN forecast training window.

Satellite altimeter data is often reprocessed as time passes in order to produce quality data sets. This means that the SLA data posted for a given date goes through multiple iterations before being finalized. More details about the altimeter reprocessing techniques for the data set used here are detailed in its user manual (E.U. Copernicus Marine Service Information, 2025a). For this data set, there is significant variability in the SLA data set for any given batch of near-real-time data for the first 6 days, counted back from the current day. For all ESNs used here, the hindcast and forecast prediction windows are 10 days long. This means that the last few days in the training period for an ESN forecast are based on data that has yet to be completely reprocessed, which could negatively impact forecast accuracy. Hindcast ESNs used here do not face this problem because their training window does not overlap with the immediate SLA reprocessing window.

#### 3.3.4 Accuracy of the lead method for float trajectory prediction

The lead method for float trajectory prediction discussed in Section 2.2.2 was used each day to estimate the next surfacing location of floats during the deployment. The FloatCast optimization was initiated from these lead prediction locations to expedite the process of giving floats new commands when they surfaced. Here, the accuracy of these predictions is assessed to understand the fidelity of the prediction model implemented.

**FIGURE 13:**
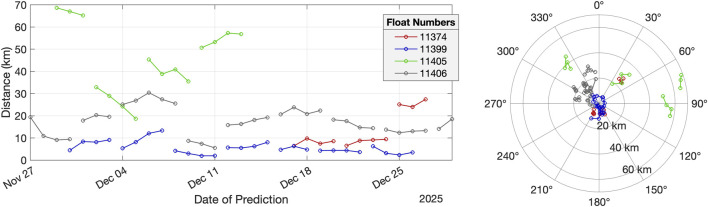
Prediction error discrepancy for the lead method for float trajectory prediction. Plotted points are connected based on the dive-cycle to which they correspond. (Left) Magnitude of the prediction error discrepancy versus time. For technical reasons floats did not always perform as expected, such as 11374 during the first part of the FloatCast control window, and 11405 going offline halfway through the control window. (Right) Magnitude and direction of prediction error discrepancy. Distance and azimuth are computed from the actual surfacing location to the predicted surfacing location. Azimuth is the clockwise angle this error line makes with north. The mean and standard deviation of the expected magnitude distance error for all floats is 18.03±15.87km .

Figure 13 shows the prediction error discrepancy for the lead method of float trajectory prediction over the duration of the FloatCast control window. Here, the prediction error discrepancy is calculated by computing the distance and azimuth from the actual surfacing location to the predicted surfacing location for each dive. Figure 13 (left) shows the magnitude of the prediction error discrepancy with respect to time. The times on the horizontal axis correspond to the date that the prediction was made. Floats performed dives of approximately 4 days in length under FloatCast, so trajectory predictions for consecutive days are typically for the same dive. The prediction error data points that correspond to the same float dives are connected together. Differences in the prediction errors for the same dive are indicative of changes in the sea surface currents used to generate each prediction. This is because the only information fed into the float prediction model that changed each day was related to surface currents. Thus, any changes in the prediction errors for the same float dive must be attributed to these differences in surface flows. As one example, float 11405 had large variability in its prediction error discrepancy for some of its dives. This could be because it spent much of the deployment in relatively strong currents from an anticyclonic eddy in the southeastern corner of the domain (Figure 10 (right)) and used shallower dive commands.

The mean and standard deviation of the magnitude of the prediction error discrepancy for floats 11374, 11399, 11405, and 11406, are 12.96±8.17 , 5.68±2.84 , 45.61±15.84 , and 17.29±6.10km , respectively. Although float 11405 had statistically the worst prediction error discrepancy, it was actually one of the success stories for FloatCast control. This float was in waters that were generally pushing it toward the region of interest. By using shallow dive commands for this float, the tradeoff was made for aggressive movement at the cost of uncertainty. In this case, although the prediction error discrepancy was quite high, the float moved much closer to the region of interest over the duration of FloatCast control. Therefore, even though the typical prediction error discrepancy values are not insignificant, this does not mean that FloatCast was ineffective. When minimizing prediction uncertainty is of greater importance than moving toward the region of interest, FloatCast settings can be modified so that more predictable commands are chosen.

Figure 13 (right) shows the magnitude of the prediction error discrepancy with the azimuth included. Interestingly, the prediction clustering shows that the changes in the surface current data over the course of a float dive do not drastically alter the expected float surfacing location. This supports our use of ESN surface flow forecasts because there is no discernible trend of float trajectory predictions improving or worsening (for each dive) as forecast flows are replaced with measured flows in the prediction model. It is not completely clear what accounts for the directional component of the prediction error discrepancy. Assuming that the FloatCast model of float movement is sufficiently accurate, it could be that the spatiotemporal resolution of the surface currents is a limiting factor. It also may be that our assumptions about the dominance of surface currents are overstated.

## 4 Discussion

This work presents FloatCast, a framework for maneuvering buoyancy-controlled ocean-profiling floats to meet observational goals. The three modules of FloatCast are the ocean surface flow forecaster, float trajectory integrator, and float dive command selector. The flow forecasting module is built around an ESN. Float trajectory prediction is handled using a Lagrangian particle model. For a given set of candidate dive commands, float trajectories are predicted based on a method that extends ocean surface velocity downward. The selection of float dive commands is based on the relative sampling performance of each set of trajectory predictions. Sampling performance is determined according to a mapping error scoring metric, which rewards command sets that are expected to help floats fully occupy the region of interest. Operationally, this metric is intended to attract floats to the region of interest and keep floats already in the region from drifting away. These FloatCast capabilities improve the value of measurements obtained from ocean-profiling floats for programs that have similar observational goals.

Simulation results from two case studies are also discussed. First, simulation results are presented from trial runs of FloatCast using historical data from floats deployed in the Gulf Stream during July 2024. The first simulation uses a purely deterministic float prediction model. Outputs are shown from each of the three FloatCast modules to provide insight into how these functionalities overlap. For the system configuration considered, it is optimal to increase the float sampling rate using shorter dives at the expense of reduced float spacing. The second simulation uses a stochastic float prediction model. The analysis shows how prediction uncertainty can be incorporated into float control decisions. Although the optimal commands from this analysis are the same as the previous case study, there is inherent value in understanding the expected performance uncertainty. This case study also demonstrates the importance of regenerating fresh trajectory predictions to limit the growth of uncertainty over time. The greedy search method for dive command selection is also shown to limit the computational demand of FloatCast.

Experimental results from the live application of FloatCast control to a float deployment in the Philippine Sea in November–December 2025 are also analyzed. For the first time, FloatCast was used for closed-loop control of a small fleet of active floats. The communication capabilities of FlowPilot (Szuts et al., 2023) were critical to this effort. The resulting float trajectories and intermediate outputs of FloatCast for this control window are also evaluated. Due to inherent systematic constraints, concrete conclusions cannot be drawn regarding the expected performance gained or lost from using the FloatCast software to control the floats. However, despite operational challenges typical of ocean-sampling missions, the initial results showed promise.

In ongoing and future work, high-fidelity simulation tools can replicate experimental configurations to help determine the expected benefit of FloatCast control. Using these tools, permutations of closed-loop control choices can be evaluated over time in the same virtual environment. This would enable us to see how the system would have evolved if different commands had been chosen at each time step—something that is not possible in the real world. The resulting sequence of ground-truth optimal commands can be compared against commands generated from closed-loop FloatCast control of floats in the virtual environment and the real world. Comparisons like this would deepen our understanding of FloatCast control performance as it relates to stochastic effects of the ocean.

Similar simulation analyses may reveal to which system components the FloatCast framework is most sensitive. Understanding this can be helpful for making targeted improvements to the system. For example, the spatiotemporal resolution of FloatCast flow predictions is fairly low, and the Lagrangian particle model used in FloatCast is based on simple assumptions. Analysis from high-fidelity simulations corroborated by experimental results can help uncover how each of these design choices impacts prediction error. Using FloatCast control at the beginning of a sampling mission when floats are very close together and already in the region of interest may be especially enlightening for these types of comparisons.

Even without the deeper simulation analysis described above, managing the Philippine Sea float deployment revealed opportunities for future work on FloatCast. Each of the three FloatCast modules can be refined: the ESNs can be re-tuned, the float particle model adjusted, and the scoring metric altered. In particular, float trajectory prediction accuracy may be improved by assimilating subsurface float velocity data into our flow forecasting model in real time. Alternatively, subsurface float velocity data can be used to train an ESN (or some other method) to predict float velocities in a data-driven way. These velocity predictions can be integrated to make float trajectory predictions that can be analyzed using existing FloatCast tools.

For future deployments, FloatCast can directly consider prediction uncertainty in its control decisions. This could further build on the methodology discussed in this paper and be used to prevent higher-risk commands from being selected. Logistically speaking, the most important next step is the full automation of the FloatCast software for real-world use. This will necessitate more robust exception-handling of live float data. Improvements can also be made to how floats are modeled when they are outside the ESN prediction domain.

## Data Availability

The raw data supporting the conclusions of this article will be made available by the authors, without undue reservation.

## Footnotes

1 For more information on the Perron-Frobenius operator, see Froyland and Padberg (2009).

2 Theoretically, geostrophic balance happens at scales larger than the Rossby radius of deformation (Ro, in space) and the inertial period ( $T_{\text{inert}}$ in time). Though both depend on latitude, representative mid-latitude values applicable here are Ro=50km and $T_{\text{inert}}=1.25\;days$ .

3 See Morris et al. (2024) for more information on operational logistics of profiling floats.

## References

[B1] Bretherton F. P. Davis R. E. Fandry C. (1976). A technique for objective analysis and design of oceanographic experiments applied to MODE-73. Deep Sea Res. Oceanogr. Abstr. 23, 559–582. 10.1016/0011-7471(76)90001-2

[B2] Chamberlain P. Talley L. D. Cornuelle B. Mazloff M. Gille S. T. (2023a). Optimizing the biogeochemical argo float distribution. J. Atmos. Ocean. Technol. 40, 1355–1379. 10.1175/JTECH-D-22-0093.1

[B3] Chamberlain P. Talley L. D. Mazloff M. Van Sebille E. Gille S. T. Tucker T. (2023b). Using existing argo trajectories to statistically predict future float positions with a transition matrix. J. Atmos. Ocean. Technol. 40, 1083–1103. 10.1175/JTECH-D-22-0070.1

[B4] Chelton D. B. deSzoeke R. A. Schlax M. G. El Naggar K. Siwertz N. (1998). Geographical variability of the first Baroclinic Rossby radius of deformation. J. Phys. Oceanogr. 28, 433–460. 10.1175/1520-0485(1998)028⟨0433:GVOTFB⟩2.0.CO:2

[B5] Choi J. g. (2016). Physical Oceanography: Geostrophic Current (Numerical).

[B6] Cucchi M. Abreu S. Ciccone G. Brunner D. Kleemann H. (2022). Hands-on reservoir computing: a tutorial for practical implementation. Neuromorphic Comput. Eng. 2, 032002. 10.1088/2634-4386/ac7db7

[B7] Cui Y. Wu R. Zhang X. Zhu Z. Liu B. Shi J. (2025). Forecasting the eddying ocean with a deep neural network. Nat. Commun. 16, 2268. 10.1038/s41467-025-57389-2 40050275 PMC11885467

[B8] E.U. Copernicus Marine Service Information (CMEMS) (2025a). Global Ocean Gridded L 4 Sea Surface Heights and Derived Variables Nrt. Mar. Data Store (MDS). 10.48670/moi-00149 [Dataset]

[B9] E.U. Copernicus Marine Service Information (CMEMS) (2025b). Global Ocean Gridded L 4 Sea Surface Heights and Derived Variables Reprocessed 1993 Ongoing. Mar. Data Store (MDS). 10.48670/moi-00148 [Dataset]

[B10] Froyland G. Padberg K. (2009). Almost-invariant sets and invariant manifolds — connecting probabilistic and geometric descriptions of coherent structures in flows. Phys. D. Nonlinear Phenom. 238, 1507–1523. 10.1016/j.physd.2009.03.002

[B11] Gaillard F. Reynaud T. Thierry V. Kolodziejczyk N. Von Schuckmann K. (2016). In situ–based reanalysis of the Global Ocean temperature and salinity with ISAS: variability of the heat content and steric height. J. Clim. 29, 1305–1323. 10.1175/JCLI-D-15-0028.1

[B12] Gill A. E. (1982). Atmosphere-Ocean dynamics. Int. Geophys. Ser. 30, 193.

[B13] Goswami D. Riggins A. Paley D. A. (2022). Data-Driven Prediction of Urban Micromobility: A Study of Dockless Electric Scooters [Applications of Control]. IEEE Control Syst. 42, 18–31. 10.1109/MCS.2022.3187328

[B14] Grossi M. D. Jegelka S. Lermusiaux P. F. Özgökmen T. M. (2025). Surface drifter trajectory prediction in the Gulf of Mexico using neural networks. Ocean. Model. 196, 102543. 10.1016/j.ocemod.2025.102543

[B15] Hassanibesheli F. Kurths J. Boers N. (2022). Long-term ENSO prediction with echo-state networks. Environ. Res. Clim. 1, 011002. 10.1088/2752-5295/ac7f4c

[B16] Holbrook N. J. Sen Gupta A. Oliver E. C. J. Hobday A. J. Benthuysen J. A. Scannell H. A. (2020). Keeping pace with marine heatwaves. Nat. Rev. Earth and Environ. 1, 482–493. 10.1038/s43017-020-0068-4

[B17] Johnson G. C. Purkey S. G. (2024). Refined estimates of global Ocean deep and abyssal decadal warming trends. Geophys. Res. Lett. 51, e2024GL111229. 10.1029/2024GL111229

[B18] Kubin E. Menna M. Mauri E. Notarstefano G. Mieruch S. Poulain P.-M. (2023). Heat content and temperature trends in the Mediterranean Sea as derived from Argo float data. Front. Mar. Sci. 10, 1271638. 10.3389/fmars.2023.1271638

[B19] Leonard N. E. Paley D. A. Lekien F. Sepulchre R. Fratantoni D. M. Davis R. E. (2007). Collective motion, sensor networks, and Ocean sampling. Proc. IEEE 95, 48–74. 10.1109/JPROC.2006.887295

[B20] Lidström S. Wickberg A. (2025). Co-environing the ocean and climate: the Argo program. Environ. Plan. E Nat. Space 8, 2002–2017. 10.1177/25148486251377704

[B21] Liu X. Dunne J. P. Drenkard E. J. Johnson G. C. (2025). Simulating Argo float trajectories and along-track physical and biogeochemical variability in the California Current System. Front. Mar. Sci. 12, 1481761. 10.3389/fmars.2025.1481761

[B22] Lukoševičius M. (2012). “A practical guide to applying echo State networks,” in Neural Networks: Tricks of the Trade. Editors Montavon G. Orr G. B. Müller K.-R. Second Edition (Berlin, Heidelberg: Springer Berlin Heidelberg), 659–686. 10.1007/978-3-642-35289-8_36

[B23] Ma B. B. Girton J. B. Dunlap J. H. Gobat J. I. Chen J. (2023). “Passive acoustic measurements on autonomous profiling floats,” in OCEANS 2023 - MTS (IEEE U.S. Gulf Coast), 1–6. 10.23919/OCEANS52994.2023.10337032

[B24] Morris T. Scanderbeg M. West-Mack D. Gourcuff C. Poffa N. Bhaskar T. V. S. U. (2024). Best practices for Core Argo floats - part 1: getting started and data considerations. Front. Mar. Sci. 11, 1358042. 10.3389/fmars.2024.1358042

[B25] Nielsen-Englyst P. Høyer J. L. Kolbe W. M. Dybkjær G. Lavergne T. Tonboe R. T. (2023). A combined sea and sea-ice surface temperature climate dataset of the Arctic, 1982–2021. Remote Sens. Environ. 284, 113331. 10.1016/j.rse.2022.113331

[B26] Paley D. A. (2007). Cooperative control of collective motion for ocean sampling with autonomous vehicles

[B27] Schaeffer P. Pujol M.-I. Veillard P. Faugere Y. Dagneaux Q. Dibarboure G. (2023). The CNES CLS 2022 mean Sea surface: short wavelength improvements from CryoSat-2 and SARAL/AltiKa high-sampled altimeter data. Remote Sens. 15, 2910. 10.3390/rs15112910

[B28] Smith R. N. Huynh V. T. (2014). Controlling buoyancy-driven profiling floats for applications in Ocean observation. IEEE J. Ocean. Eng. 39, 571–586. 10.1109/JOE.2013.2261895

[B29] Sun C. Song M. Cai D. Zhang B. Hong S. Li H. (2024). A systematic review of Echo State networks from design to application. IEEE Trans. Artif. Intell. 5, 23–37. 10.1109/TAI.2022.3225780

[B30] Szuts Z. Harrison T. Curtin T. Kirby B. Ma B. (2023). “FlowPilot: shoreside autonomy for profiling floats,” in *OCEANS 2023 - MTS/IEEE U.S. Gulf Coast* (Biloxi, MS, USA: IEEE), 1–6. 10.23919/OCEANS52994.2023.10337384

[B31] Tolone J. Harrison T. Curtin T. Szuts Z. Paley D. A. (2025). “A modular control aid for profiling floats with a Gulf stream case Study,” in OCEANS 2025 - Great Lakes, 1–10. 10.23919/OCEANS59106.2025.11245129

[B32] Troesch M. Chien S. Chao Y. Farrara J. Girton J. Dunlap J. (2018). Autonomous control of marine floats in the presence of dynamic, uncertain ocean currents. Robotics Aut. Syst. 108, 100–114. 10.1016/j.robot.2018.04.004

[B33] Van Sebille E. Aliani S. Law K. L. Maximenko N. Alsina J. M. Bagaev A. (2020). The physical oceanography of the transport of floating marine debris. Environ. Res. Lett. 15, 023003. 10.1088/1748-9326/ab6d7d

[B34] Wang T. Liu Z. Du Y. (2024). A synthetic autonomous profiling float array in a Lagrangian particle tracking system. Acta Oceanol. Sin. 43, 34–46. 10.1007/s13131-024-2395-7

[B35] Wiggert M. Doshi M. Lermusiaux P. F. Tomlin C. J. (2022). “Navigating underactuated agents by hitchhiking forecast flows,” in *2022 IEEE 61st Conference on Decision and Control (CDC)* (Cancun, Mexico: IEEE), 2417–2424. 10.1109/CDC51059.2022.9992375

[B36] Wong A. P. S. Wijffels S. E. Riser S. C. Pouliquen S. Hosoda S. Roemmich D. (2020). Argo data 1999–2019: two million temperature-salinity profiles and subsurface velocity observations from a global array of profiling floats. Front. Mar. Sci. 7, 700. 10.3389/fmars.2020.00700

